# Bi_1−x_La_x_CuSeO as New Tunable Full Solar Light Active Photocatalysts

**DOI:** 10.1038/srep24620

**Published:** 2016-04-20

**Authors:** Huanchun Wang, Shun Li, Yaochun Liu, Jinxuan Ding, Yuan-Hua Lin, Haomin Xu, Ben Xu, Ce-Wen Nan

**Affiliations:** 1 State Key Laboratory of New Ceramics and Fine Processing, School of Materials Science and Engineering, Tsinghua University, Beijing 100084, People’s Republic of China; 2 High-Tech Institute of Xi’an, Xi’an, Shanxi 710025, People’s Republic of China; 3 School of Materials Science and Engineering, University of Science and Technology Beijing, Beijing 100083, People’s Republic of China

## Abstract

Photocatalysis is attracting enormous interest driven by the great promise of addressing current energy and environmental crises by converting solar light directly into chemical energy. However, efficiently harvesting solar energy for photocatalysis remains a pressing challenge, and the charge kinetics and mechanism of the photocatalytic process is far from being well understood. Here we report a new full solar spectrum driven photocatalyst in the system of a layered oxyselenide BiCuSeO with good photocatalytic activity for degradation of organic pollutants and chemical stability under light irradiation, and the photocatalytic performance of BiCuSeO can be further improved by band gap engineering with introduction of La. Our measurements and density-functional-theory calculations reveal that the effective mass and mobility of the carriers in BiCuSeO can be tuned by the La-doping, which are responsible for the tunable photocatalytic activity. Our findings may offer new perspectives for understanding the mechanism of photocatalysis through modulating the charge mobility and the effective mass of carriers and provide a guidance for designing efficient photocatalyts.

Photocatalysts can directly convert solar energy into chemical energy, representing a relatively simple and environment-friendly route for renewable energy generation and environmental remediation issues^1^,^2^. Presently, the most predominant and extensively studied photocatalysts are wide band gap semiconductors (e.g. TiO_2_, SrTiO_3_ and SnO_2_, etc.) that can only absorb photons in the UV or near-UV regime, which accounts for merely 5% of the total solar energy irradiation and thus renders the overall process impractical. As about 45% of sun light is in the visible light wavelength region (Vis, 400–800 nm) and 50% is in the form of near infrared light (NIR, >800 nm), the central issue of current research is to extend the light absorption of photocatalysts. To this end, tremendous attempts, including doping various transition metals and nonmetals^3^,^4^,^5^,^6^,^7^, exploring alternative narrow band gap semiconductors^8^,^9^,^10^, and loading visible light sensitizers such as plasmonic metal nanostructures^11^,^12^,^13^, have been under taken toward effective sunlight harvesting and conversion. While a plethora of research has focused on developing visible light responsive photocatalysts, the use of long wavelength NIR lights are still scarce. Up to now, only few NIR light active photocatalysts have been reported such as Cu_2_(OH)PO_4_^14^, Bi_2_WO_6_^15^ and WS_2_^16^, as well as up-conversion material systems^17^,^18^,^19^,^20^. It is highly desirable and yet challenging to develop novel photocatalysts capable of using the entire solar spectrum with high efficiency and chemical stability^21^.

It is generally believed that the capabilities of a photocatalyst in light absorption, charge generation and transportation highly depend on its electronic structures. Recently, great efforts and progress have been made on probing and understanding the energy band structure and charge kinetics in various semiconductor photocatalysts to rationalize the link between the photocatalytic activity and electronic characteristics^6^,^22^,^23^,^24^,^25^. In particular, the effective mass and/or charge mobility play a significant role on the separation and transfer of photogenerated carriers^26^,^27^,^28^,^29^, thus it is highly crucial to unveil kinetics of these charge transfer processes for fundamental understanding of the mechanism and further developing efficient photocatalysts. A great deal of theoretical effort has been spent on clarifying the effect of effective masses of electrons and holes along different directions on the photocatalytic performance by employing density functional theory (DFT) calculations^29^,^30^,^31^. For example, Zhang *et al.*^31^ reported that anatase phase TiO_2_ possess the lightest average effective mass of photogenerated electrons and holes as compared to rutile and brookite, thus resulting in the lowest recombination rate of photogenerated charge carriers. To the best of our knowledge, however, studies on this topic still remain at the theoretical level, and all reported works are lack of direct evidence that can determine or estimate the effective mass of carriers in an experimental way, which will provide critical support for the theoretical investigations. This highly important yet unresolved issue motivated our current research.

In this work, we demonstrate a new Bi-based layered structured BiCuSeO oxyselenides with good photocatalytic activity and chemical stability in the full solar light spectrum for degradation of organic contaminants (Congo Red being used as model pollutants) in aqueous solution, and present a comprehensive study on the role of effective mass on the photocatalytic properties in a series of Bi_1−x_La_x_CuSeO samples (La doping being employed for tailoring the energy band structure of BiCuSeO) both experimentally based on Hall measurements on the bulk samples and theoretically by DFT calculations. Our results reveal that La-doping can effectively tune the carrier mobility by modulating the primary valence band and effective mass at Fermi level. Photocatalytic activity of the as-prepared samples displays strong correlation to the carrier mobility, and the improvement of carrier mobility is in favour of photocatalytic performance. To the best of our knowledge, our findings constitute the first experimental evidence of determination of effective mass/charge mobility in a photocatalyst (in the case of Bi_1−x_La_x_CuSeO), supported by theoretical simulations. This investigation will provide some new perspectives into understanding the relationship of photocatalytic activity and charge kinetics in semiconductor photocatalytic systems.

## Results and Discussion

**Figure 1:**
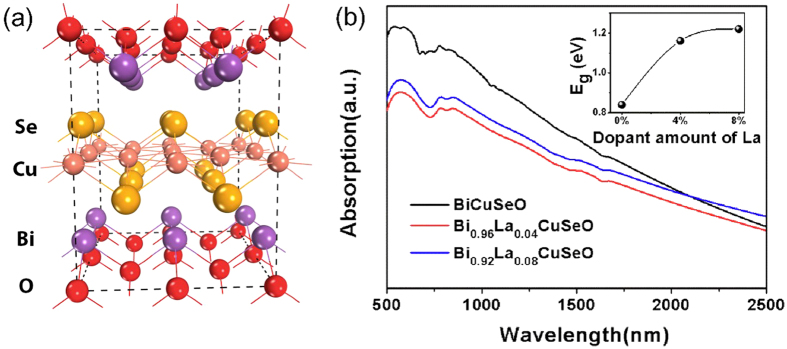
(**a**) The crystalline structure of La-doped BiCuSeO used in DFT calculations. (**b**) UV-Vis-NIR absorbance spectra of Bi_1−x_La_x_CuSeO (x = 0, 0.04, 0.08) powder samples. Insert shows the variation of bandgaps with different La doping content estimated by Kubelka-Munk transformation.

Oxyselenide compound BiCuSeO has been extensively investigated as a promising oxide-based thermoelectric material in the moderate temperature range in recent years triggered by Zhao’s work in 2010^32^. A variety of methods have been employed to tune the electrical and thermal properties, including doping to optimize charge carrier concentration^33^,^34^,^35^,^36^,^37^ and tune the transport properties of carriers^38^, introducing Cu deficiencies^39^ and band gap tuning to enhance electrical conductivity^40^, and texturing to improve the mobility of carriers^41^. The crystal structure of BiCuSeO belongs to the ZrSiCuAs-type structure with the tetragonal *P*4/*nmm* space group that constitutes the alternately stacked (Bi_2_O_2_)^2+^ insulating layers and (Cu_2_Se_2_)^2−^ conductive layers along the *c*-axis of the tetragonal cell, as illustrated in Fig. 1a. Motivated by their unique layered structure, favorable transport properties of carriers and tunable narrow band gap, Bi_1−x_La_x_CuSeO samples with different compositions (x = 0, 0.04 and 0.08) in powdered form were prepared (see Methods) to test their potential utilization as photocatalysts. Powder X-ray diffraction (Fig. S1, Supplementary Information) reveals that all diffraction peaks can be well indexed to the pure phase of BiCuSeO (JCPDS no. 45–0296), without impurity peaks appearing in all as-prepared samples. The lattice constants *a* and *c* increase with increasing La doping content from 0 to 0.08, indicating that La^3+^ is successfully incorporated into the BiCuSeO lattice. The scanning electron microscopy (SEM) image (Fig. S2a, Supplementary Information) demonstrates that the crushed BiCuSeO powders are consist of irregular shaped particles with dimensions from tens to hundreds of nanometers. The polycrystalline nature of the BiCuSeO sample was further confirmed by high resolution transmission electron microscopy (HRTEM). Agglomerated nanocrystals with grain sizes ranging from 5 to 10 nm can be observed to form polycrystalline particles with amorphous-like boundary (Fig. S2b, Supplementary Information). However, individual well-crystallized nanocrystals were also noticed, as exemplified in Fig. S2c in the Supplementary information.

UV-Vis-NIR diffuse reflection spectrum (DRS) was measured to characterize the absorption feature of all the samples. As shown in Fig. 1b, pure BiCuSeO shows impressive absorption in the entire solar spectrum covering UV, Vis, NIR and even part of IR regions. Compared to the pure BiCuSeO, La ion substitution slightly alters the absorption characteristics of BiCuSeO, evidenced by its lower absorption below 2000 nm. The band gap width of all samples was calculated according to Kubelka-Munk method^42^. As shown in the inset of Fig. 1b, the bandgap increases along with the increase of La doping content. Band gap energy of 0.84 eV was estimated for pure BiCuSeO, consistent with reported value^40^, while 1.15 eV and 1.22 eV for the ones doped with 4% and 8% of La, respectively. Some minor peaks appeared in the absportion curves of the samples with La-doping and show the shift, which should be ascribed to induced trap levels and variation of chemical bonds of Bi-O and La-O. As La doped in BiCuSeO, our calculation and experimental results reveal that it will lead to the broadening of band gap, and the electronic structure of band gap will influence the position of peaks.

**Figure 2:**
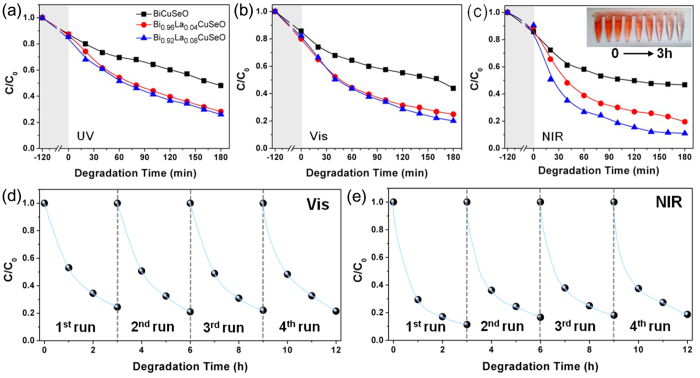
Photocatalytic degradation of Congo Red solution in the presence of Bi_1−x_La_x_CuSeO (x = 0, 0.04, 0.08) powders under irradiation of (**a**) UV (λ = 365 ± 5 nm), (**b**) visible (420 nm < λ < 780 nm) and (**c**) near-infrared light (800 nm < λ < 1100 nm). Cycling runs using Bi_0.92_La_0.08_CuSeO powders under (**d**) visible and (**e**) near-infrared light irradiation.

The photocatalytic activity of a series of Bi_1−x_La_x_CuSeO powders was evaluated by monitoring the decomposition of the model pollutants Congo Red (CR) aqueous solution under UV, visible and NIR light, respectively. A 5W LED with emission wavelength of 365 ± 5 nm was used as the UV light source, and a 300 W xenon lamp with 420 nm and 800 nm filters was used as visible and NIR light source, respectively. As shown in Fig. 2a–c, all the samples exhibit good broad-spectrum responsive photocatalytic activity. Pristine BiCuSeO displays inherent photocatalytic activity in UV, visible and NIR light region, and about 45%, 49% and 44% of CR solution was photodegraded in 180 min, respectively. The photocatalytic activity is comparable with the previous works in Cu_2_(OH)PO_4_^14^ and Bi_2_WO_6_^15^. Fascinatingly, although the band gap of BiCuSeO broaden slightly after doping with La, Bi_1−x_La_x_CuSeO (x = 0.04 and 0.08) powders show more preferable photocatalytic activity in the whole solar light region, and the degradation rate enhances with increasing La content. With the assistant of Bi_0.92_La_0.08_CuSeO, nearly 70%, 75% and 90% of CR solution was degraded under UV, visible and NIR light, respectively, within the same reaction time.

The photocatalytic process basically involves adsorption-degradation-releasing process, and the adsorbed organic pollutions on the surface of photocatalysts is prerequisite for photo-induced reaction. BET analysis demonstrated that the specific surface areas of crushed powder samples are 2.852 m^2^·g^−1^, 2.478 m^2^·g^−1^ and 2.512 m^2^·g^−1^ for Bi_1−x_La_x_CuSeO with x = 0, 0.04 and 0.08, respectively, indicating negligible influence of the surface area on the photocatalytic performance. To rule out the possibility of adsorption rather than degradation of CR solution in the presence of Bi_1−x_La_x_CuSeO photocatalysts, Fourier transform infrared (FTIR) spectra of the CR powder, as well as the Bi_0.92_La_0.08_CuSeO photocatalyst were characterized, which gives us an insight into the change of surface functional group before and after going through a complete photocatalytic process. As shown in Fig. S3 in the Supplementary Information, the peaks attributed to CR did not appear on Bi_0.92_La_0.08_CuSeO after photocatalytic reaction, indicating that the decrease of concentration of the CR solution should result from decomposition instead of adsorption.

To assess the chemical stability of Bi_1−x_La_x_CuSeO as photocatalysts under light illumination, XRD measurements were performed on pure BiCuSeO and Bi_0.92_La_0.08_CuSeO before and after photocatalytic reaction. The XRD patterns (Fig. S4, Supplementary Information) indicate that the crystal structure of both of two samples remain unchanged during the photocatalytic process, thus demonstrating excellent structural stability of the BiCuSeO-based photocatalytsts. Cycling runs for photodegradation of CR solution under visible and NIR light were also carried out, as displayed in Fig. 2d,e. Bi_0.92_La_0.08_CuSeO maintains comparable activity even after four repeated runs. The robustness of photocatalytic performance suggests that this material might be practically useful as a stable photocatalyst.

**Figure 3:**
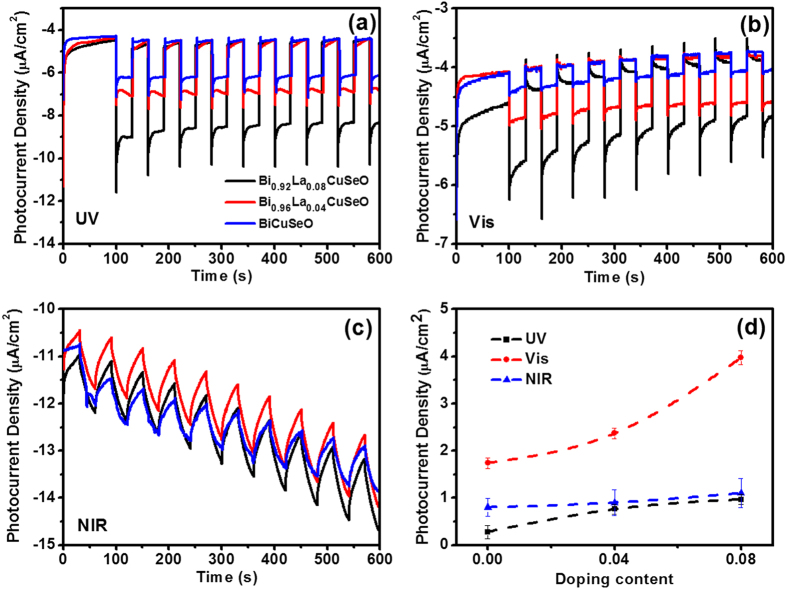
Transient photocurrent curves (at 0 V versus RHE) of the bulk Bi_1−x_La_x_CuSeO (x = 0, 0.04, 0.08) electrode under (**a**) UV, (**b**) visible and (**c**) near infrared light illumination. (**d**) Comparison of photocurrent density of various samples under illumination of different light source.

Although direct correlations between photocurrent and photocatalytic activity is still absence, close relationship of them are reported and adopted to characterize the property of photocatalysts^43^,^44^,^45^. Generally, larger photocurrent density indicates that more electron and hole pairs are generated under illumination that will take part in the redox reaction occurring on the active site of the surface, thus leading to superior photocatalytic performance. As Bi_1−x_La_x_CuSeO is effective in full solar light region for degradation of azo dye, and improvement of photocatalytic performance due to the introduction of La was confirmed, further investigation was explored using photoelectrochemical (PEC) method to get a deeper understanding of the effect of La doping. PEC measurements were performed on electrochemical work station with standard three electrodes system in 0.5 M Na_2_SO_4_ electrolyte and Bi_1−x_La_x_CuSeO ceramic plate (thickness of ca. 360 μm) as working electrode. Pristine and La-doped BiCuSeO photoelectrodes demonstrate similar photocurrent response under full spectrum region (Fig. 3). BiCuSeO photoanode shows photocurrent response as high as 1.74 μA/cm^2^ and 0.28 μA/cm^2^ under irradiation of UV and visible light, respectively. Correspondingly, the photoresponse is more intense with photocurrent density of 3.97 μA/cm^2^ and 0.97 μA/cm^2^ demonstrated by 8% La-doped specimen. Such enhancement was sustainable during the on-off switch of light illumination. Although no statured current was obtained under the irradiation of NIR light, the photocurrent reveals similar trend in pristine and La-doped BiCuSeO photoanodes. Obviously, La-doped BiCuSeO samples exhibit superior PEC property than pure BiCuSeO under the same incident intensity in the whole solar spectrum. It is also worth noting that the photoelectrodes are made of thick ceramic plate (c.a. 360 μm), thus the photoresponse could be further enhanced by decreasing the thickness or preparing thin film BiCuSeO materials for solar to chemical/electrical conversions.

Generally, photocatalysis is considered to involve three processes, i.e., i) illumination inducing a transition of electrons from valence band (VB) to conduction band (CB), leaving holes at the top of VB; ii) transport and recombination of electron-hole pairs and iii) redox reaction on the surface of photocatalyst^2^. In the present work, the photocatalysts were used without any modification of surface, so the surface chemistry properties are supposed to be identical to one another macroscopically. Therefore, the generation and transport properties of the photogenerated electron-hole pairs should be responsible for the improvement of photocatalytic property with the introduction of La.

According to previous reports, the transport property of charge carriers is drastically influenced by the effective mass^26^,^29^,^46^,^47^. Smaller value of hole effective mass (*m*_h_^*^) usually leads to a greater mobility, therefore resulting in much more straight forward transfer to the surface due to the field built in crystal intrinsically, and thus suppressing the charge recombination^47^. However, the effective mass and mobility of carriers are anisotropic in photocatalysts, as explored theoretically in a great number of semiconductors such as Ag_3_PO_4_^29^, TiO_2_^48^ and ZnO^49^ *etc.*, using DFT calculations. The difference of photocatalytic activity on specific exposed facets was suspected to be closely related to relevant effective mass/charge mobility. In this sense, it is reasonable to enhance the photocatalytic property of photocatalysts through optimizing the carrier effective mass/mobility in bulk or nanostructures. However, experimental routes of directly measure or estimate the effective mass/mobility in photocatalyst have not been addressed yet, and detailed study of the transport property induced by doping is still lacking. In the flowing we will discuss the relationships of effective mass and the photocatalytic performance in Bi_1−x_La_x_CuSeO both theoretically and experimentally.

**Figure 4:**
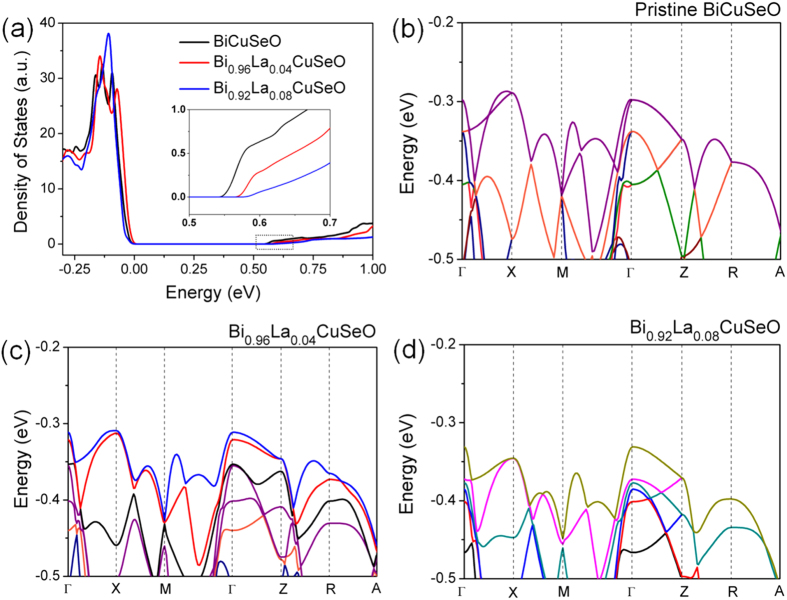
(**a**) Total density of state near the Fermi level and (**b**–**d**) the valence band maximum of Bi_1−x_La_x_CuSeO with increasing La content.

BiCuSeO is reported as a multiband semiconductor^34^. The top of the valence band (L band) consists of a hole pocket (located on Γ-M line of the Brillouin zone), which has been proven to be a heavy band. The secondary valence band maximum observed on the Γ-X line and at the Z points is a light band (Σ band). The band structures of Bi_1−x_La_x_CuSeO were investigated by theoretical calculation using DFT plus on-site repulsion U (DFT+U) in the Vienna ab initio Simulation Package (VASP). The effective masses of hole located at the top of light band and heavy band are 0.81 *m*_*e*_ and 1.91 *m*_*e*_ (where *m*_*e*_ is the free-electron mass), respectively, and the light band locates slightly lower than the heavy band. That is consistent with reported feature^50^. The broadening of band gap with the introduction of La was confirmed in plots of total density of states (Fig. 4a). The increasing tendency of band gap is consistent with the experimental value estimated by the absorption spectra (Fig. 1b). The calculated band gap of pristine BiCuSeO is 0.54 eV, which is smaller than experimental value. According to the calculated results, the VBM (or CBM) shifts to more positive induced by the doping of La, resulting in broadening of band gap (Fig. S6, Supplementary Information). The conduction band minimum is at the Z point, and the valence band maximum is on the M–G line, indicating that BiCuSeO is an indirect band gap semiconductor.

Light band is considered to be more beneficial to the transport of carriers and was utilize to tune the mobility of carriers through band engineering. Moreover, the effective mass of holes dependent not only on the primary valence band, but also on the secondary valence band maximum. Revealed by DFT calculation, the primary valence band of BiCuSeO is heavy band, and VBM located on Γ-M line of the Brillouin zone. With the introduction of La, the secondary valence band maximum shift less positive than the primary, leading to the approaching of the two. With 8% of La doping, the light band locates slightly higher than the heavy band, and VBM locates at Γ band which is light band) That means light band turns into valence band maximum, and carriers can be supposed to be have higher mobility. Moreover, the change of valence band maximum from X to Γ band leads to the transform of indirect band gap to direct band gap semiconductor induced by La doping, for the bottom of conduction band located at Γ band. Direct band gap semiconductors are considered to have higher absorption coefficient which should have higher conversion rate of incident light.

To get further insights into the effect of energy band structure and carrier property of BiCuSeO by the introduction of La, the carrier concentration, effective masses and mobility were measured and estimated experimentally. Intrinsic carrier concentration at room temperature was measured according to Hall effect over bulk Bi_1−x_La_x_CuSeO samples. The carrier concentration decreased from 1.82 × 10^19 ^cm^−3^ in pristine BiCuSeO to 1.45 × 10^19 ^cm^−3^ and 9.71 × 10^18 ^cm^−3^ in Bi_0.96_La_0.04_CuSeO and Bi_0.92_La_0.08_CuSeO, respectively. Obviously, the substitution of Bi ion with La will form LaCuSeO crystalline, leading to decreased carrier concentration as compared to the parent phase.

**Figure 5:**
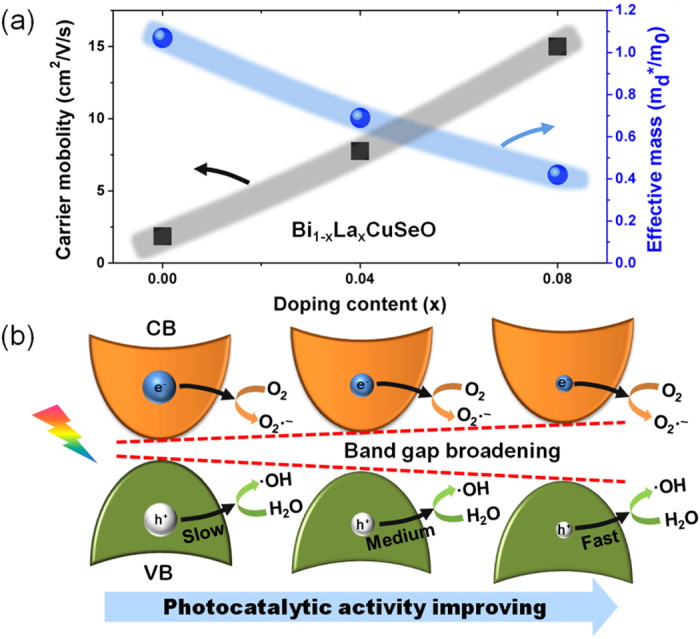
(**a**) Carrier mobility and effective mass of carrier measured according to Hall effect. (**b**) Schematic illustration of the enhanced photocatalytic and charge transfer mechanism. The sizes of sphere areas represent the differences in effective mass of electrons (blue) and holes (white).

The effective masses of carriers were estimated using Pisarenko relation^51^, which was typically used in the field of thermoelectrics:


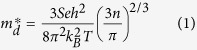


where *S*, *k*_B_, *T*, *e*, *h*, *m*_d_^*^ and *n* are Seebeck coefficient, Boltzmann constant, absolute temperature, carrier charge, Plank constant, effective mass of hole at Fermi level, and the hole concentration, respectively. As shown in Fig. 5a, the effective mass of holes decreases with the increase of La-doping content, while the mobility shows a reverse trend. The mobility is 1.85 cm^2^/V·s for pristine BiCuSeO, and increases to 7.75 cm^2^/V·s and 15 cm^2^/V·s in Bi_0.96_La_0.04_CuSeO and Bi_0.92_La_0.08_CuSeO, respectively.

The mechanism of the enhanced photocatalytic activity is tentatively proposed in Fig. 5b. A higher mobility of holes favors a faster and more efficient transport of hole to the surface for oxidation reaction, thus resulting in superior photocatalytic/photoelectrochemical performance. As discussed before, with smaller effective mass (higher mobility) of holes, La doped BiCuSeO show stronger photocurrent density as well as higher photocatalytic activity of degrading CR solution than pristine one in UV, visible and NIR light under the same intensity of irradiation. This can be considered as direct evidence of more efficient migration of carriers to surface over La doped BiCuSeO than pure sample. Different from introducing impurity band to narrow the band gap and to tune the photocatalytic property in typical semiconductor photocatalysts, substituted Bi with La in BiCuSeO did not form new impurity band as confirmed by theoretical simulations. This should be ascribed to the good compatibility of La ion in the parent crystal, for LaCuSeO has the same space group and close crystal lattice^52^. With this methodology, the energy band structure and carrier property can be easily modulated without introduction of impurity band which usually act as the recombination center of photogenerated carriers. Our work provides a direct evidence for exploiting efficient photocatalyst for photocatalysis application in full solar light region. Further studies are underway to apply this strategy in other semiconductor photocatalyst systems.

Photocatalytic degradation of dyes is associated with the formation of active oxidation species (AOS). In the present study, CR solution can be photo-degraded by various AOS, for example, •O_2_^−^ and •OH. To further clarify the mechanism, we carried out photoluminescence (PL) measurements using terephthalic acid as a fluorescence probe, as reported previously^47^. Our results showed that fluorescence peak at 420 nm can be detected due to formation of 2-hydroxyterephthalic acid (Fig. S5), as a result of the reaction between terephthalic acid and •OH. Therefore, we conclude that the formation of •OH formed by the photo-excited holes and H_2_O under illumination are mainly responsible for the oxidation of the CR dye solution. These results are consistent with the Hall measurements discussed above, indicating that the holes are dominant under both light and dark.

In summary, we have reported layered oxychalcogenides Bi_1−x_La_x_CuSeO as new photocatalysts in the entire solar spectrum region for the first time. Photodegradation of Congo Red aqueous solution without any co-catalysts implied good photocatalytic activity of BiCuSeO-based narrow bandgap semiconductors. Partial substitution of Bi with La leads to further improved photocatalytic performance by tuning the carrier mobility and modulating the energy band structure. We found out that the photocatalytic activity enhanced with decreasing effective mass of carriers, since it is advantageous for the transfer of photogenerated holes to the surface reaction sites during the photocatalytic process. These results may present a new research direction of investigating charge kinetics which is a key factor of determining the photocatalytic activity and provide new perspectives for deep understanding of the mechanism of photocatalysis in semiconductor material systems.

## Methods

### Synthesis of Bi_1−x_La_x_CuSeO

All Bi_1−x_La_x_CuSeO samples were made from chemical-reagent-grade Bi_2_O_3_, La_2_O_3_, Cu, Bi and Se powders by solid state reaction method, as reported in our previous work^53^. Typically, stoichiometric mixture of the powders was ball-milled in an argon atmosphere for 4 h first. Subsequently, the obtained powders were cold-pressed into pellets with diameter of 20 mm and sealed in an evacuated quartz tube, which was firstly heated at 573 K for 5 h and then 973 K for 10 h. The as-prepared pellets were crushed into powders followed by ball milling for hours in vacuum.

### Characterization

Powder X-ray diffraction (XRD) was performed on a Bruker D8-Advance diffractometer using monochromatized Cu K*α* (*λ* = 0.15418 nm) radiation with scanning speed of 3°/min. A field emission scanning electron microscope (JSM-7001F, JEOL) operating at a 5 kV was used to characterize the morphology of the samples. The transmission electron microscopic (TEM) images were collected with a JEOL JEM-2100 electron microscopy at an acceleration voltage of 200 kV. UV-Vis-NIR diffuser reflectance (DRS) measurements were carried out on UV/Vis/NIR spectrometer (PerkinElmer, Lambda 950). Fourier transform infrared spectroscopy (FTIR) of the samples was recorded on a Fourier Transform Infrared Spectrometer (VERTEX 70 V, Bruker).

### Photocatalytic Activity Measurement

The photocatalytic activity of the as-prepared Bi_1−x_ La_x_CuSeO powder samples was evaluated by photodegrading Congo Red (CR, 100 mg/L) aqueous solution. Typically, 0.16 g photocatalyst powder was dispersed into 80 mL CR solution and stirred in dark for 2 h in advance to reach the adsorption-desorption equilibrium between the photocatalysts and organic dye molecules. Cooling-water bath and magnetic stirring were hold continuously to prevent thermal effect during the degradation process and to keep the uniformity. A 5 W LED with emission wavelength of 365 ± 5 nm was used as the UV light source, and a 300 W xenon lamp with 420 nm and 800 nm cut-off filters was used as visible and NIR light source, respectively. The incident light source was placed above the aqueous solution vertically with illumination intensity of about 78 mW/cm^2^, 132 mW/cm^2^, and 473 mW/cm^2^ for UV, visible and NIR lights respectively at upper surface of the solution. At regular time intervals, 3 mL suspension was collected and centrifuged, and the residual CR concentration in the supernatant was analyzed by UV-vis spectrophotometer (UV-3100, Hitachi). For cycling runs, photocatalyst was centrifuged and collected followed by washing with deionized water and drying at 70 °C. The photocatalytic measurement was carried out under the same conditions as mentioned before.

### Photoelectrochemical Testing

Photocurrent measurement was carried out with a standard three electrode system on an electrochemical workstation (CHI 660, ChenHua, Shanghai). Ag/AgCl and Pt wires were used as reference electrode and counter electrode respectively in Na_2_SO_4_ solution (0.5 mol. L^−1^) as electrolyte. Working electrode was made by pasting silver on one side of the sintered and polished Bi_1−x_La_x_CuSeO ceramic plate with thickness of ca. 350 μm. The photocurrent of the Bi_1−x_La_x_CuSeO photoanode under UV, visible and NIR lights irradiation were recorded at a bias of 0 V versus the reference electrode.

### Measurement of Carrier Concentration and Mobility

For carrier concentration and mobility measurement, bulk Bi_1−x_La_x_CuSeO samples were sintered by spark plasma sintering (SPS) under a pressure of 50 MPa at 873 K for 8 min using as-prepared powder. The obtained SPS-ed pellets were cut along the radial direction of the disk sample into bars with dimensions of about 15 mm × 3 mm × 3 mm. The Seebeck coefficient were measured using a thermoelectric measuring apparatus (ZEM-2, ULVAC-RIKO, Japan) under a helium atmosphere from 300 to 873 K.

### Computational modelling

Density Functional Theory (DFT) based on the first principles is used as the calculation method in the Vienna ab initio Simulation Package (VASP). For more independent evidence of how the band structure changes with La content, we performed first principle calculation using the Perdew-Burke-Ernzerhof (PBE)+U, where the Coulomb (U) and exchange parameters (J) for La 4*f*-electrons and Cu 3*d*-electrons were chosen according to previous reports^54^,^55^,^56^.

For BiCuSeO, a non-parabolic isotropic band is assumed for the electronic structure around the valence band maximum (VBM). For a non-parabolic band, the definition for the effective mass, containing the first derivative *dε*/*dk*, is found to be more convenient than which contains the second derivative *d*^2^*ε*/*d*^2^*k*. The effective mass can be written as:


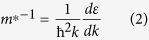


where m^*^ denotes the effective mass of hole around the VBM, 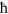 is the reduced Planck constant, and k is the modulus of wave vector.

In our calculations, we used a plane-wave energy cutoff of 600 eV and an energy convergence criterion of 10^−8 ^eV. A k-mesh of 6 × 6 × 4 was adapted for the band calculations because 3 × 2 × 2 superlattice was chosen. And La fractions of 4.17% and 8.33% were used in the calculation of Bi_1−x_La_x_CuSeO to represents 4% and 8% doping, respectively.

## Additional Information

**How to cite this article**: Wang, H. *et al.* Bi_1–x_La_x_CuSeO as New Tunable Full Solar Light Active Photocatalysts. *Sci. Rep.* **6**, 24620; doi: 10.1038/srep24620 (2016).

## Supplementary Material

Supplementary Information
