# Supplementary material for: Bi1−xLaxCuSeO as New Tunable Full Solar Light Active Photocatalysts
Source: Sci Rep. 2016 Apr 20;6:24620. doi: 10.1038/srep24620 (PMC4837348; doi:10.1038/srep24620)
Supplement: Supplementary Information [file srep24620-s1.pdf]

Supplementary Information

$Bi_{1-x}La_xCuSeO$ as New Tunable Full Solar Light Active Photoatalysts

Huanchun Wang,[1,2] Shun Li,[3] Yaochun Liu,[1,3] Jinxuan Ding,[1] Yuanhua Lin,[1, a)],

Haomin Xu, [1] Ben Xu[1] and Cewen Nan[1]

[1]*State Key Laboratory of New Ceramics and Fine Processing, School of Materials Science and Engineering, Tsinghua University, Beijing 100084, People's Republic of China*

[2]*High-Tech Institute of Xi'an, Xi'an, Shanxi 710025, People's Republic of China*

[3]*School of Materials Science and Engineering, University of Science and Technology Beijing, Beijing 100083, People's Republic of China*

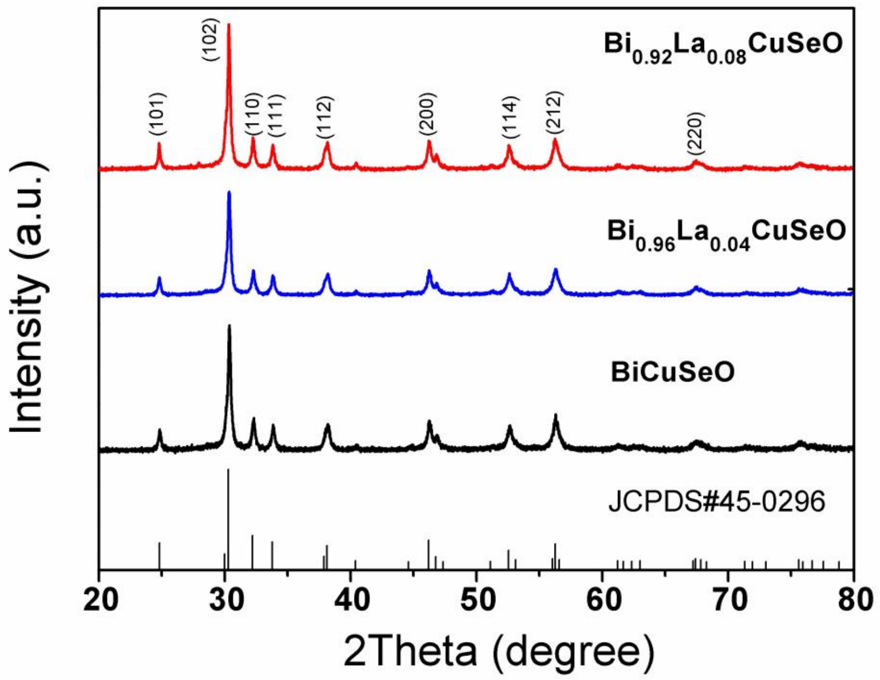

Figure S1. Powder XRD patterns of $Bi_{1-x}La_xCuSeO$ (x = 0.00, 0.04, 0.08).

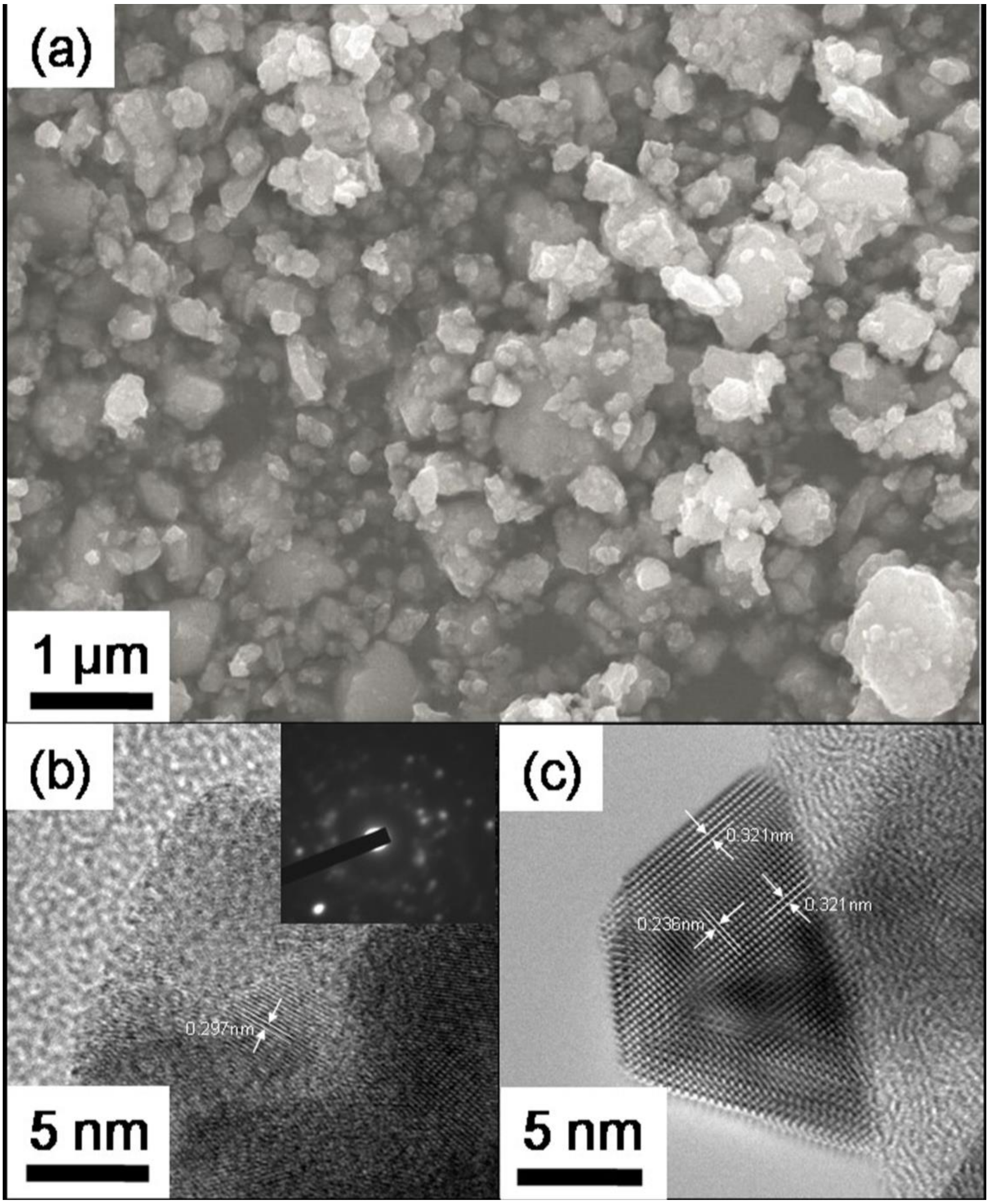

Figure S2. (a) SEM and (b and c) HRTEM image of the BiCuSeO powder. Inset of (b) shows the SAED patterns.

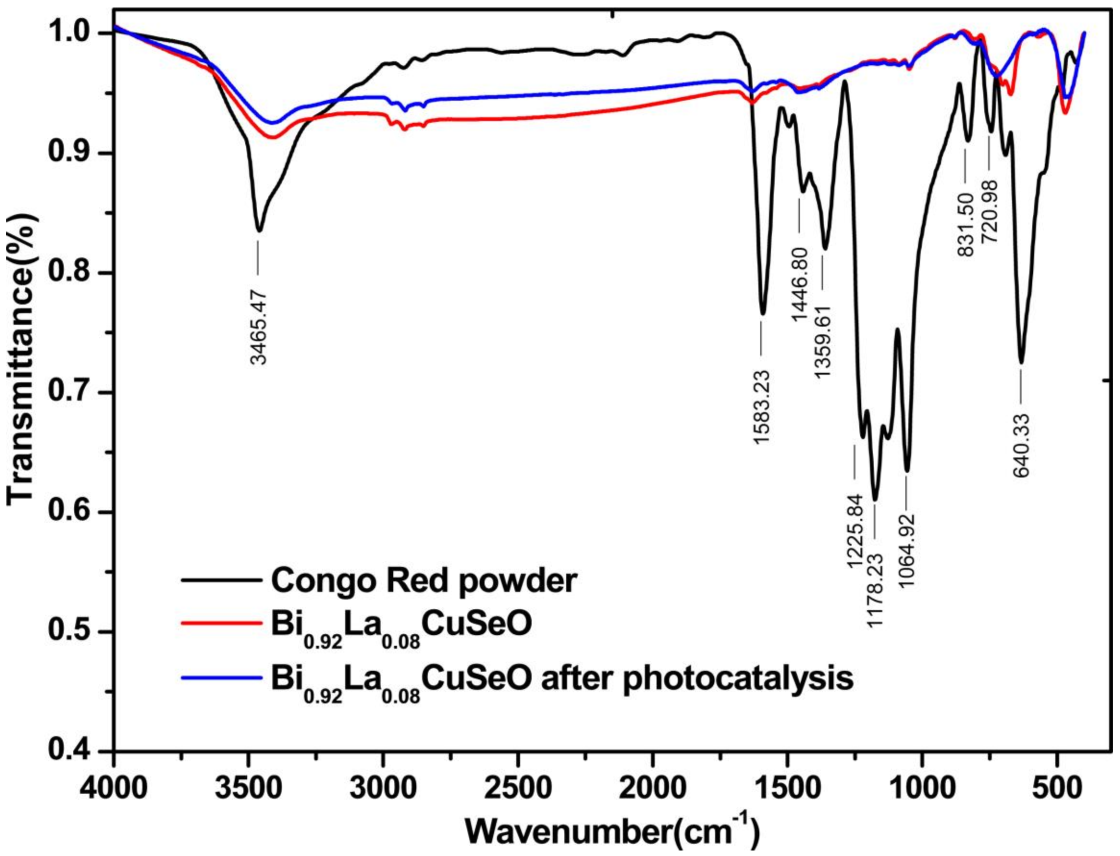

Figure S3. FTIR spectra of Congo Red powder and $Bi_{0.92}La_{0.08}CuSeO$ sample before and after photocatalytic process.

Congo Red azo dyes show absorption peaks at low wave numbers range from 500 cm^{-1} to 1700 cm^{-1} due to the vibration of covalent bonds. The peaks at 1583.23 cm^{-1} belongs to the symmetrical stretching vibration of -N=N-, which is the characteristic peak of azo dyes. The bond between C and S was distinguished from the peaks of 1064.92 cm^{-1} and 640.33 cm^{-1}, which results from the stretching vibration of -C-S-. However, these characteristic absorption vanished over BiCuSeO photocatalysts after photocatalytic process, indicating that no detectable Congo Red molecule was attached to the surface of the photocatalyst. It is also worth noting that BiCuSeO shows almost the same absorptive character to pristine photocatalyst after going throuth the photocatalytic process. These results demonstrate that the concentration decline of Congo Red aqueous is not caused by physical absorption of dye on photocatalyst.

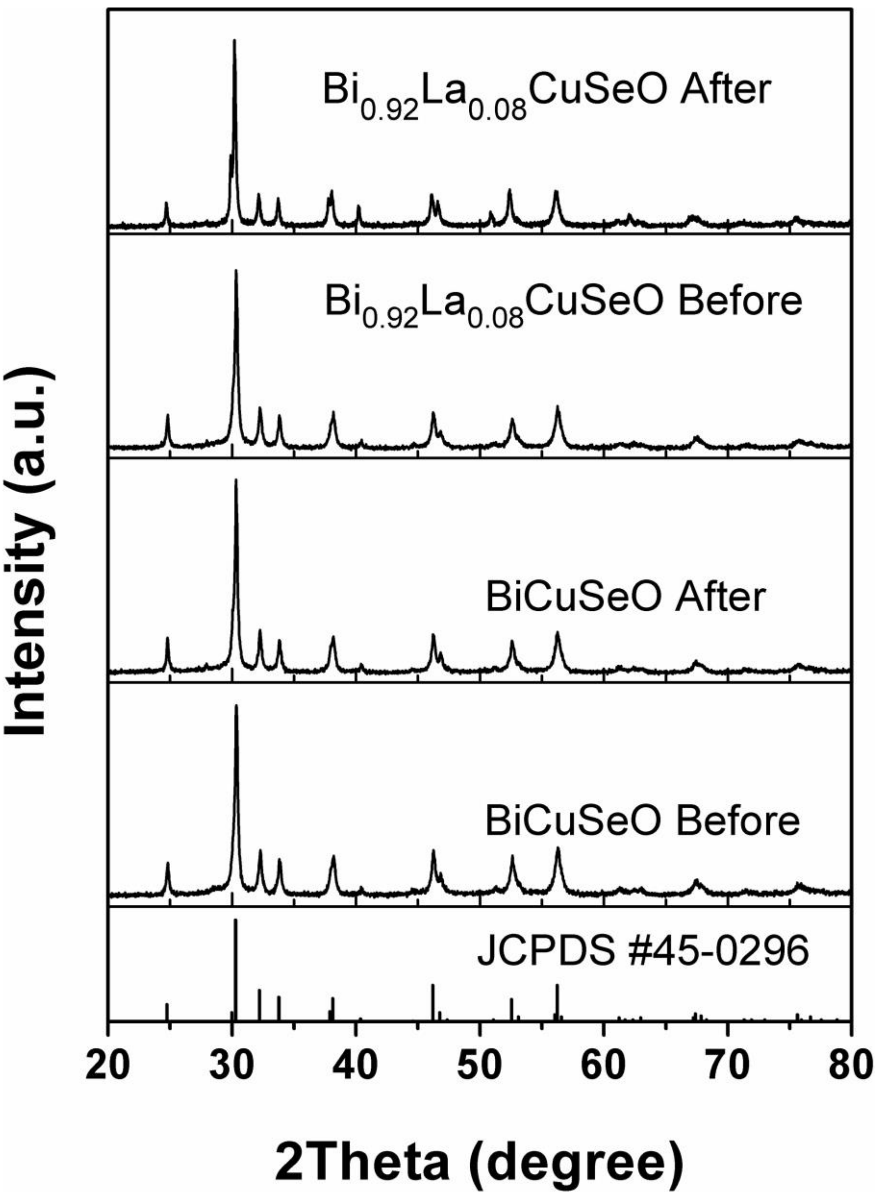

Figure S4. XRD patterns of BiCuSeO and $Bi_{0.92}La_{0.08}CuSeO$ samples before and after photocatalytic reaction.

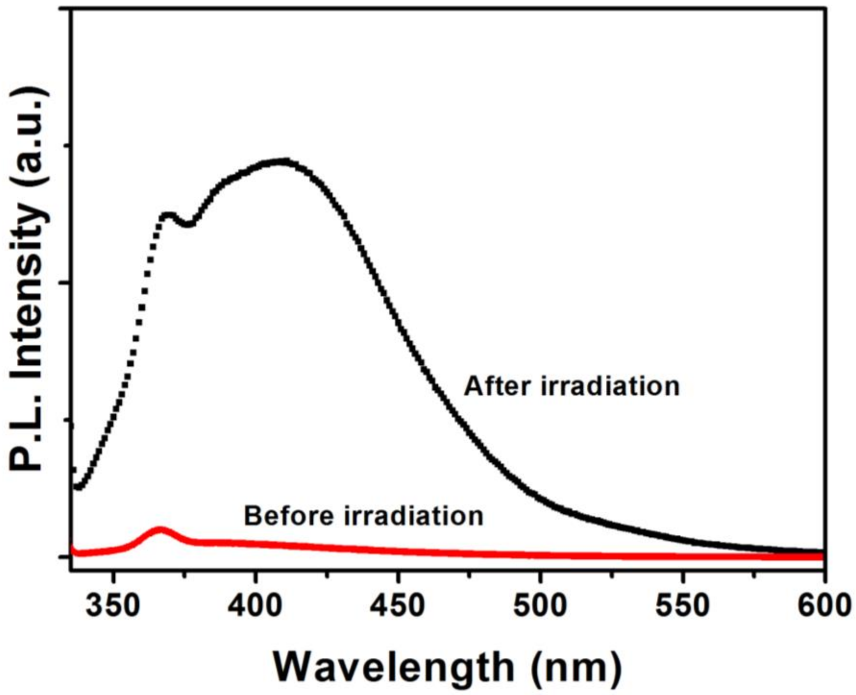

Figure S5. Fluorescence spectral changes observed during visible illumination of BiCuSeO in terephthalic acid solution (4×10^{-4} M, excitation at 315 nm). Photoluminescence measurement was carried out using solution with terephthalic acid (0.166 g) and NaOH (0.16 g) in 200 mL deionized water. 0.5 g of BiCuSeO powder was added and stirred for 60min. After 60 min visible light irradiation, 5 mL mixture was drawn and centrifuged for PL measurement.

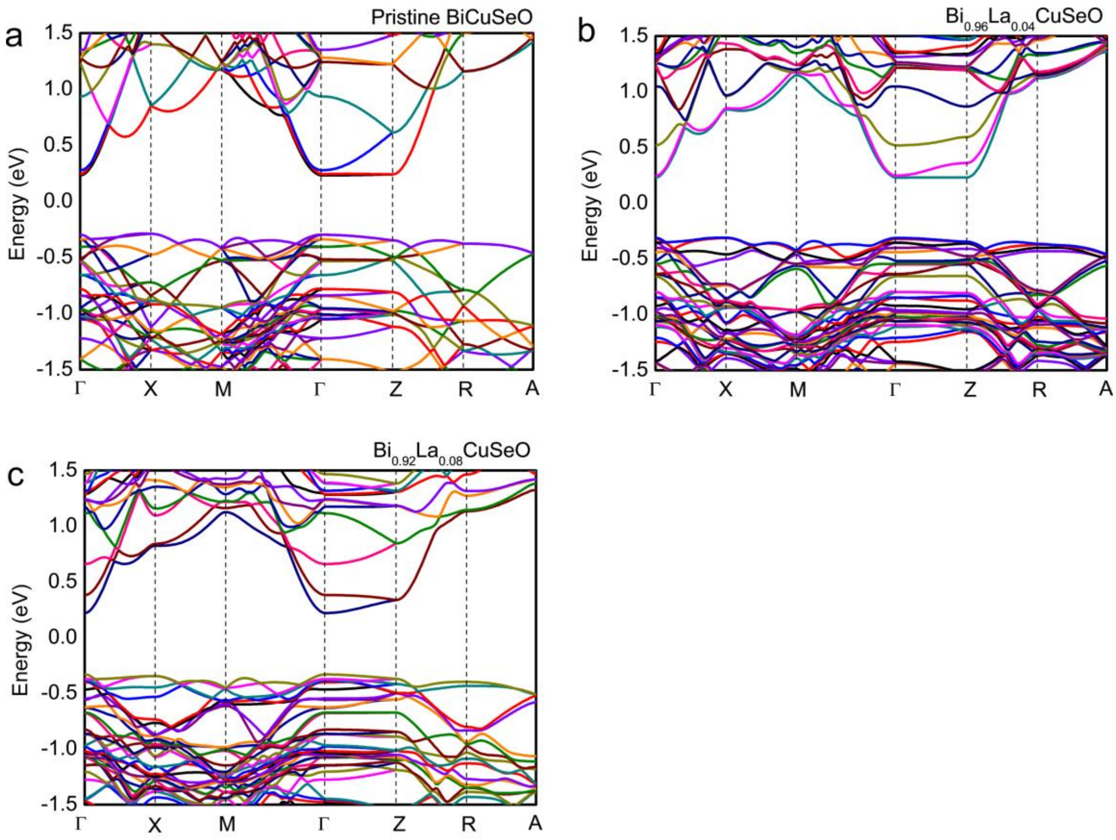

Figure S6. Calculated energy band structure of $Bi_{1-x}La_xCuSeO$ along with the increase of La content..

Density Functional Theory (DFT) based on the first principles was used as the calculation method in the Vienna ab initio Simulation Package (VASP). Perdew-Burke-Ernzerhof (PBE)+*U*, where the Coulomb (*U*) and exchange parameters (*J*) for La 4f-electrons and Cu 3d-electrons were chosen. A k-mesh of 6×6×4 was adapted for the band calculations because 3×2×2 superlattice was chosen. And La fractions of 4.17% and 8.33% were used in the calculation of $Bi_{1-x}La_xCuSeO$ to represent 4% and 8% doping, respectively.
